# Clinical trials for stem cell therapies

**DOI:** 10.1186/1741-7015-9-52

**Published:** 2011-05-10

**Authors:** Alan Trounson, Rahul G Thakar, Geoff Lomax, Don Gibbons

**Affiliations:** 1 California Institute for Regenerative Medicine, 210 King Street, San Francisco, CA 9107, USA

## Abstract

In recent years, clinical trials with stem cells have taken the emerging field in many new directions. While numerous teams continue to refine and expand the role of bone marrow and cord blood stem cells for their vanguard uses in blood and immune disorders, many others are looking to expand the uses of the various types of stem cells found in bone marrow and cord blood, in particular mesenchymal stem cells, to uses beyond those that could be corrected by replacing cells in their own lineage. Early results from these trials have produced mixed results often showing minor or transitory improvements that may be attributed to extracellular factors. More research teams are accelerating the use of other types of adult stem cells, in particular neural stem cells for diseases where beneficial outcome could result from either in-lineage cell replacement or extracellular factors. At the same time, the first three trials using cells derived from pluripotent cells have begun.

## Review

The rapid advance of stem cell clinical trials for a broad spectrum of conditions warrants an update of the review by Trounson (2009) [1]. There has been a rapid surge in clinical trials involving stem cell therapies over the last two to three years and those trials are establishing the clinical pathways for an emergent new medicine. These early trials are showing roles for stem cells both in replacing damaged tissue as well as in providing extracellular factors that can promote endogenous cellular salvage and replenishment.

### Bone marrow, umbilical cord blood, placental and mesenchymal stem cells

There are many studies involving autologous therapies and some allogenic therapies, based on the recovery of mobilized bone marrow cells, including mesenchymal stem cells (MSCs) and adipose derived stem cells that also include the stromal or adherent cell type that has an MSC phenotype. Human umbilical cord blood cells have been used in a large number of trials for paraplegia, ataxia, multiple sclerosis, amyotrophic lateral sclerosis, cerebrovascular disease, multiple system atrophy, motor neuron disease, among other indications, without severe immunological response [2]. Placenta-derived stem cells are being considered for similar uses and are in Phase III clinical trial for critical limb ischemia by Israel's Pluristem Therapeutics.

A significant proportion of clinical studies that are underway involve bone marrow and cord blood stem cells for blood and immune disorders [3] and cancers. Several of those are now considered applicable for patient treatments beyond the need for regulated clinical trials. We have chosen to concentrate on the emerging therapeutics that broadly involves a wide range of cell types in clinical trials registered on the National Institutes of Health's clinical trials web site.

**Figure 1:**
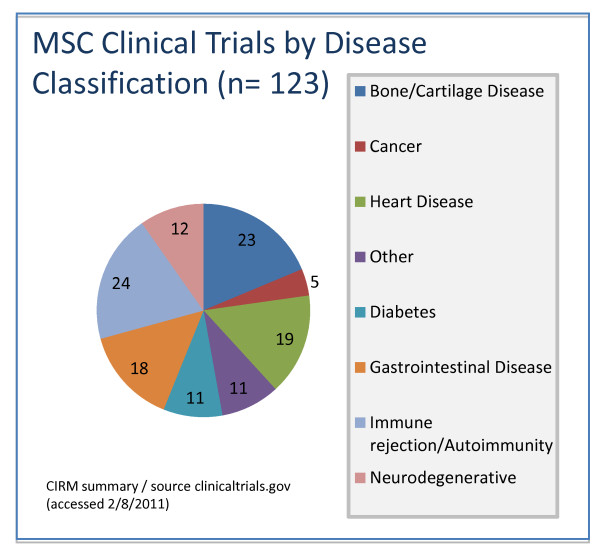
**Diseases being addressed using mesenchymal stem cells (MSC) for clinical trials (n = number of trials)**.

**Figure 2:**
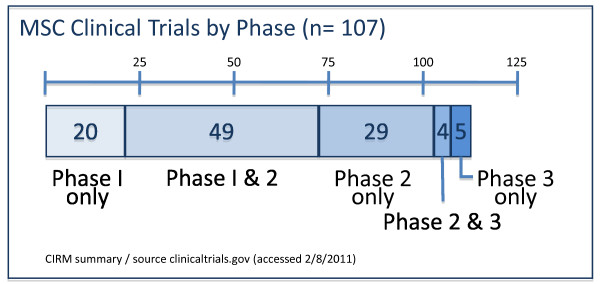
**Mesenchymal stem cell (MSC) clinical trials by clinical phase (n = number of trials)**.

MSCs are a stromal cell type and the current definition of MSCs includes plastic adherence in cell culture, specific surface antigen expression (CD105(+)/CD90(+)/CD73(+), CD34(-)/CD45(-)/CD11b(-) or CD14(-)/CD19(-) or CD79α(-)/HLA-DR1(-)), and multi-lineage *in vitro *differentiation potential (osteogenic, chondrogenic, and adipogenic) [4]. The public clinical trials database http://clinicaltrials.gov shows 123 clinical trials using MSCs for a very wide range of therapeutic applications (Figure 1), the majority of which are in Phase I (safety studies), Phase II (proof of concept for efficacy in human patients), or a mixture of PhaseI/II studies (Figure 2). This includes bone and cartilage repair, cell types into which MSCs readily differentiate, and immune conditions such as graft versus host disease and autoimmune conditions that utilize the MSC's immune suppressive properties. Expectations for patient benefits are high in these therapeutic applications. Nevertheless, there are many prospective applications where the mechanism of action is not obvious and some concerns have been expressed about the likelihood of long-term benefit of these applications. In the case of allogenic MSCs, delivery to an inflamed site can result in gain of immune potency with accelerated damage due to a heightened immune-mediated inflammatory response [5].

### Cardiac repair

The use of patient's own bone marrow aspirates, hematopoietic stem cells and MSCs, for heart muscle tissue repair can be puzzling because these cells do not normally contribute to the cardiac lineage types that are desired. There is some preclinical data in support of umbilical cord blood for improved cardiac function for myocardial infarction [6] but sustained patient recovery has not been clearly demonstrated. It has been shown, that these blood and stromal cells may, *in vitro*, form sarcomeric structures typical of cardioimyocytes with expression of some genes expected of these cell types: atrial natriuretic peptide (ANP), brain natriuretic peptide (BNP), and contractile proteins including myosin heavy chain, myosin light chain, and alpha actin [7]. There is little evidence, however, of myocardial regeneration *in vivo*, despite 3% to 4% (range 2% to 7%) improvement in the global left ventricular function and cardiac ejection fraction (contractility), but not left ventricular remodeling, in meta-analyses following intracoronary infusions for myocardial infarction [7-9]. In comparative studies of MSCs and cardiac (c-kit+) stem cells and cardiosphere derived cells, [10] the cardiomyogenic differentiation capacity was clearly more effective with the cardiac derived cells than with MSCs. There is a distinct possibility that procedure-related variables influence the positive outcomes for patients. So there is a need to optimize treatment timing, cell type and dose, and delivery methods. Also research needs to determine the potential tropic influence of stem cell secretions or cytokines released at the site of injury and the degree of cardio-repair that may be clinically relevant [11]. A recent study by Lee and colleagues at Harvard found a subset of marrow cells that was able to stimulate endogenous adult cardiac stem cells, offering a possible mechanism for the effect seen [12]. It is possible that protein-based rather than cell-based therapies may evolve from these studies. It is pretty clear that for the ventricular remodeling required, more effective cell types with significant populating capacity will be needed to replace the severely damaged infarct area of the heart.

### Neurological applications

Studies involving umbilical cord blood for neurological indications have been promoted as a result of preclinical data on the apparent formation of neurons *in vitro *[13] but there is little evidence of their transdifferentiation to functional neurons or glial cells *in vivo*. Mobilized peripheral blood cells (CD34+) delivered into the femoral artery have been used in safety studies for chronic spinal cord injury without adverse effects but with very little evidence of efficacy in follow up [14].

Clinical trials involving use of MSCs for the treatment of neurological disorders is also relatively common (Figure 1), despite little evidence for their conversion to neural cells *in vivo*. Autologous MSCs isolated from bone marrow and injected intrathecally into spinal cord cerebrospinal fluid, allowing access to the brain and spinal column, can be accomplished safely in patients with multiple sclerosis and amyotrophic lateral sclerosis (ALS). Karussis et al. (2010) [15] provided some evidence of immunomodulatory effects of MSCs within 24 hours of intrathecal injection but claims of ferumoxides labeled MSCs persisting after three to six months was less persuasive.

### Immunological applications

Multiple sclerosis is currently treated with steroids, immunomodulating agents, immunosuppression and humanized monoclonal antibodies (*Natalizumab*) and more recently by immunosuppression followed by transplantation of autologous CD34+ hematopoietic stem cells (HSCs) aimed to reconstitute the immune system following the removal of active autoreactive T cells. This would enable the establishment of tolerance to autoantigens and a period of remission in diseases such as multiple sclerosis. With more than 400 patients treated in Phase I/II trials there is benefit seen in inflammatory parameters and disease progression, particularly in rapidly evolving severe multiple sclerosis [16]. Whether these early improvements in clinical parameters of disability will translate into long-term benefits and sustained remission is as yet unclear. There are strong recommendations to undertake randomized comparative trials of HSC transplantation versus non-transplantation with a large target patient population to validate any benefit for HSC therapy [17]. Given the need for strong immunosuppression as part of the strategy, the benefits need to substantially outweigh the risks inherent in the treatment. There have been some studies using allogenic HSCs but none are currently reported active. This approach is only considered in advanced nonmalignant disease because of unfavorable risk to benefit ratio [17]. Further study is warranted for HSC therapy in these patients.

Systemic sclerosis, systemic lupus erythematosus and Crohn's Disease as well as multiple sclerosis, are major disease targets for multinational randomized clinical trials. Improvements are observed in dermal fibrosis and pulmonary dysfunction in systemic sclerosis patients following lymphoablative conditioning and HSC therapy up to 8 years [18]. There are also trials evaluating HSC therapy in rheumatoid arthritis and juvenile idiopathic arthritis [19]. These approaches all focus on transient depletion of active immune cell numbers followed by qualitative changes in the immune cell repertoire that enables the resetting of a modified adaptive immune system that is tolerant to self-antigens, previously targeted in autoimmune disease. While present clinical trial approaches using autologous HSCs will be informative, it remains speculative whether long-term remission will be achieved in the variety of diseases under examination and improvements in therapeutic strategies are expected to evolve in time. The potential use of MSCs in resetting immune homeostasis as a therapeutic approach is being explored because of their ability for cytoprotection and immunosuppression. However, their long-term usefulness and exact role in the treatment of autoimmune diseases remains to be determined.

Chronic Graft Versus Host Disease (GVHD) has also been a target for HSC and MSC cell therapies and is usually observed after allogenic HSC or tissue transplants. This very serious condition may manifest in the peripheral or central nervous systems, and in multiple organs of the body [20]. At least ten clinical trials with MSC have been reported with mixed results, but many show a significant level of positive response. One company, Osiris, has completed patient enrollment in Phase 3 trials for steroid refractory acute GVHD and for newly diagnosed acute GVHD [21].

### Genetic blood diseases

HSC therapies are in clinical trials for genetic diseases such as sickle cell disease and β-thalassemia. In sickle cell disease, curative high levels of T-cell chimerism (>50%) using HLA-matched sibling allogenic CD34+ HSC transplantation can be achieved without myloablation [22]. New developments in stem cell gene therapy offer a potentially safer therapy for sickle cell disease in the future [23].

Long-term mixed chimerism with allogenic HSCs can be achieved in β-thalassemia but it is recommended that the donor chimerism be >25% for robust therapeutic effects in these patients [24]. However, gene therapy models involving the transduction of CD34+ HSCs with lentiviral vectors indicate that only 10% to 15% chimerism of functional thalassemic cells can be achieved which is below the therapeutically curative level [24]. Even the recovery of CD34+ cells for gene therapy, using granulocyte-colony stimulating factor (G-CSF) stem cell mobilization may be harmful in β-thalassemia patients, and may cause severe side effects in sickle cell anemia patients. This needs careful evaluation and additional consideration to minimize these adverse risks [25].

Allogenic HSC therapy in cases of inherited genetic disease may be associated with death or severe complications after transplantation making autologous gene therapy for blood and immune disease an important strategy. Boztug et al [26], have shown that HSC gene therapy for Wiskott-Aldrich syndrome, a severe X-linked recessive immunodeficiency disorder, can be largely corrected by autologous HSC gene therapy. CD34+ cells from two patients were transduced with a retroviral vector incorporating a construct expressing the correct gene (*WASP*) after transient mylosuppression with busulfan. Stable chimerism of 9% and 20% of donor hematopoietic progenitors were sufficient to effect correction of the primary disease phenotype, including hemorrhagic diathesis, eczema, autoimmunity, and severe infection. These types of clinical studies lay the foundation for stem cell gene therapy in human disease. This will likely include the potential cure of HIV/AIDS by targeted disruption of the CCR5 gene in autologous CD34+ HSCs [27].

### Adipose stem cells

Adipose stem cells are plentiful and relatively easily accessed. They have been shown to be useful for soft tissue repair [28]. They consist of adipose derived stem cells (ASCs) (CD31-/CD34+/CD45-/CD90+/CD105-/CD146-), endothelial progenitor cells and pericytes. Autologous ASCs and the stromal vascular fractions are being used for soft tissue engineering with a range of scaffolds, particularly for breast augmentation, fistulas in Crohn's disease and tissue damaged by radiation [28].

In addition to soft tissue repair, ASCs are also in clinical trial for myocardial infarction and graft versus host disease, with outcomes equivalent to MSCs [29]. They have also been used in clinical trials for tracheomediastinal fistula, Calvarial bone defect, skin ulcer and stress induced urinary incontinence.

The relative advantage of ASCs over MSCs remains to be determined for the variety of applications envisaged and further studies may demonstrate the merits of ASCs. Meanwhile, soft tissue repair and fistula repair will remain a primary application of ASCs for the immediate future.

### Endothelial stem cells

Endothelial progenitor cells (CD34+/CD133+/KDR+ or VEGFRII+) may be sourced from several sources including bone marrow, umbilical cord blood and adipose tissue. They are effective in the stimulation of angiogenesis and in clinical studies requiring revascularization and remodeling of collaterals in atherosclerotic cardiovascular disease. The result desired is the regeneration of damaged tissues, preventing amputation of ischemic limbs and other areas, and recovery after myocardial infarction. While efficacy in preclinical trials and safety in Phase I studies has been demonstrated, unequivocal evidence for patient benefit in placebo-controlled trials has not been obtained [30]. The role of endothelial progenitor cells (EPCs) in neoangiogenesis of plexiform lesions remains uncertain and there is continuing debate about the function of EPCs in the regenerative processes that are the target of EPC therapy. These matters require careful consideration in future clinical trials [31].

### Pancreatic β islet cells

Transplantation of pancreatic β Islet cells has been recently reviewed by Matsumoto [32]. Approximately 70% of Type I diabetes patients can achieve insulin independence but may have difficulty in maintaining this. They also have problems due to immunosuppression and generally there is a shortage of donors (patients need multiple donors). Xenotransplants of pig islets using encapsulation to address immune rejection is moving towards the clinic but concerns still exist for transmission of porcine endogenous retrovirus. The use of embryonic stem cell derived β Islets in special subcutaneous capsules that induce minimal fibrosis may evolve into clinical trials shortly [33].

### Neural stem cells

Neural stem cells (NSCs) may be sourced from the fetal, neonatal or adult brain. They self renew and differentiate to neurons, astrocytes and oligodendrocytes and are used in a variety of indications (Table 1). Clinical trials have been undertaken for the use of fetal neural stem cells for lysosomal storage diseases. Children with advanced stage Batten's disease (neuronal ceroid lipofuscinosis) tolerated high doses of NSCs in multiple sites in the brain in Phase I studies. The transplanted cells provide widespread global replacement enzyme, renewal for cell replacement and bystander neuroprotection [34]. The Californian company StemCells Inc. embarked on a second safety and efficacy study in children with less advanced Batten's disease using CD133+ cell culture expanded NSCs, but discontinued the study because of failure to enroll patients meeting study criteria. The company is also carrying out a Phase 1 clinical trial using fetal neural stem cell brain transplantation for Pelizaeus-Merzbacher disease (PMD), a mylination disorder that affects male children. Preclinical trials showed NSCs produce oligodendrocytes that remylinate neurons affected by the mutated gene for PMD.

**Table 1:** Neural stem cell (NSC) clinical trials underway

NSC CLINICAL TRIALS		
*Regenerative, Cell Replacement*		
**StemCells Inc., CA**	**HuCNS-SC^® ^(fetal derived human NSCs)**	
Phase I - completed	Batten's Disease (NCL)	USA
Phase Ib	Discontinued for lack of enrollment	
Phase I	Pelizaeus-Merzbacher Disease (PMD)	USA
Phase I/II	Chronic Spinal Cord Injury	Switzerland
**NeuroGeneration, CA**	**Autologous NSC-derived Neurons**	
Phase I - completed	Advanced Parkinson's Disease	USA
Phase II - clinical hold		
**Neuralstem Inc., MD**	**Fetal derived hu spinal cord SCs**	
**Phase I**	ALS (Lou Gehrig's Disease)	USA
**ReNeuron, UK**	**ReN001 Immortalized huNSCs**	
Phase I	Stroke	UK
		
***Targeted Delivery of Therapeutics***		
**City of Hope, CA**	**HB1.F3.CD Immortalized hu NSCS**	
Phase I	Recurrent High Grade Glioma	USA

Fetal NSCs are also being used for treatment of disabled ischemic stroke patients by the company ReNeuron in the UK. The NSCs have a conditional form of the oncogene encoding c-Myc under the control of an estradiol receptor that activates propogation for manufacture. Patients are transplanted with these NSCs 6 to 24 months after stroke using a straight-forward neurosurgical implantation into the brain. The NSCs express several trophic and pro-angiogenic factors that promote revascularization that may be important in ischemic stroke. The NSCs also have immunosuppressive properties that are anti-inflammatory that would aid tissue repair but they are not a persistent graft.

Studies utilizing NSCs from StemCells Inc. for chronic thoracic spinal cord injury are beginning clinical trial in Switzerland in 2011. The NSCs are injected into the spinal cord and migrate to the area of injury to form neurons and oligodendrocytes, critical for remylinating damaged neuronal axons for recovery of nerve function.

Fetal NSC preparations are in clinical trial by the company Neuralstem for the treatment of ALS (Lou Gehrig's disease). The NSCs are injected into multiple (five to ten) grey matter sites of the lumbar region of the spinal cord. The first six non-ambulatory patients showed no adverse effects of NSC engraftment. The aim is to protect healthy neural cells and repair those that have ceased communication with the patient's muscles and return ambulatory function.

Autologous NSCs obtained from patient brain biopsies have been used to treat Parkinson's disease by the company NeuroGeneration Inc., which has trial sites in California (University of California Los Angeles and Cedars-Sinai Medical Center), Italy (University of Milan) and Estonia (University of Tallin). Biopsied brain tissue is cultured *in vitro *for several months and the expanded neural stem cells differentiated into neurons, astrocytes and oligodendrocytes. These include GABAnergic (60%) and dopaminergic (15%) neurons and the mixed neurons and glia are implanted at multiple sites in the post-commissural putamen. Patients showed some motor recovery (not always sustained) and increased dopamine uptake in the transplanted putamen and clinical benefits that persist [35]. Further Phase II studies are presently on hold while manufacturing methods are established.

NSCs are also entering clinical trials for targeting the destruction of inoperable gliobastoma. NSCs home to tumors and scientists at the City of Hope, California are genetically modifying the NSCs so they produce a pro-drug activating enzyme (cytosine deaminase) that converts a non-toxic prodrug (5-Fluorocytosine, 5-FC) to a cytotoxic anticancer drug (5-Fluorouracil, 5-FU). The high local cytotoxicity will destroy gioblastomas. This is a very aggressive disease and patients are under treatment in the initial Phase I/II study.

### Limbal stem cells

Corneal disease is the second most common cause of blindness. Corneal epithelial stem cells are located at the basal layer of the limbus epithelium and provide for replacement of corneal epithelial cells that are lost or damaged. Limbal cell deficiency can be treated with transplanted limbal stem cells taken as a small biopsy and expanded *ex vivo*. Patients treated with expanded autologous limbal stem cells transplanted on human amniotic membrane had stable corneal epithelium reconstruction in all their eyes with improvement in visual acuity in the majority [36]. This appears to be a safe and effective way of restoring vision in limbal cell deficiency.

### Myoblasts

Regeneration of skeletal muscle in cases of muscular dystrophy depends on satellite cells or myogenic progenitors that are localized between the basal lamina and muscle fiber membrane [37]. Transplant trials of satellite cells or expanded myocytes, injected into muscles of patients with muscular dystrophy were shown to be safe and in some cases new dystrophin production was observed but clinical benefits were not demonstrated [35]. The problem appears to be in the need for massive numbers of injections because satellite cells distribute at local injection sites, with rapid cell loss. Also, immune responses were seen even with compatible cells, resulting in patients requiring immunosuppression [38].

The use of myoblasts for cardiac repair has been disappointing because skeletal muscle doesn't integrate functionally with cardiomyocytes, leading to a high incidence of arrhythmias [37].

### Hepatocytes

Hepatocyte transplantation is currently most successful for liver based metabolic disorders, for example to replace a deficient enzyme. This includes familial hyper-cholesterolemia, where autologous hepatocytes transduced with the low-density lipoprotein (LDL) receptor gene showed engraftment and 20% reduction in LDL cholesterol in three of five patients [39]. Allogenic hepatocyte transplantation has also been undertaken with some partial success for metabolic disorders with a few reports of long-term function of transplanted hepatocytes [39,40]. Hepatocytes are usually injected into the portal venous system and engraftment is most common in the liver or spleen.

### Pluripotent stem cells

Human embryonic stem cells (hESCs) have begun to make their way through to Phase I clinical trials (Table 2) with Geron's oligodendrocyte precursor cells derived from hESCs leading the field for safety studies on thoracic spinal cord injury. In this study, the patients need to have documented evidence of functionally complete spinal cord injury at the T3 to T10 spinal segments. The oligodendrocyte progenitors are grafted into the spinal cord at the site of injury with short-term mild immunosuppression. The company plans to extend the indication to high cervical injury, which is far more common, after the completion of the initial safety studies. The studies were originally placed on hold while the company addressed the occurrence of micro-cysts in animal transplants and screened cell line products for freedom of this characteristic.

**Table 2:** Pluripotent stem cell clinical trials (USA)

Trial sponsor	Disease target	Cell therapy
**Geron Inc**. Phase I: 10 patients enrolled 2010-12	Complete subacute thoracic spinal cord injuries. T3 to T10 segments between seven and 14 days after injury	Human embryonic stem cell derived Oligodendrocyte progenitor cells (GRNOPC1)
**Advanced Cell Technologies (ACT)** Phase I/II: 12 patients Enrolled 2011	Stargardt's Macular Dystrophy (juvenile macular degeneration)	Retinal Pigment Epithelium derived from human embryonic stem cells
**Advanced Cell Technologies (ACT)** Phase I/II: 12 patients Enrolled 2011-12	Age-related Macular Degeneration	Retinal Pigment Epithelium derived from human embryonic stem cells
**California Stem Cell (CSC)** Phase I Currently on hold 2011	Spinal muscular atrophy (SMA) Type 1	Human motor neuron progenitor cells derived from human embryonic stem cells

The company Advanced Cell Technology (ACT, CA and MA) has Phase I/II approval for clinical trials on Stargardt's Macular Dystrophy, which is a condition of blindness that arises through a photoreceptor cell protein anomaly that causes degeneration of the underlying retinal epithelium monolayer and subsequent loss of the photoreceptor cells. They have derived pigmented epithelial progenitor cells that may be injected under the photoreceptor cells to redevelop the polarized retinal epithelium monolayer. Since the primary defect is in the photoreceptor cells, it is possible that the new retinal epithelium will be lost in time and require repeat grafting. Short-term immunosuppression is to be used for grafts of allogenic retinal progenitors to the eye, even though this is considered an immune privileged site.

ACT also has approval for use of the same cells for a Phase I/II study of dry macular degeneration. This a leading cause of loss of central vision in persons older than 55 years of age. The retinal progenitors should distribute to areas of retinal degeneration and potentially correct the loss of vision.

## Conclusions

Clinical trials on the use of stem cells are underway for a wide variety of conditions and there is an emphasis on the use of bone marrow, hematopoietic (mobilized and recovered in blood and umbilical cord blood) and mesenchymal stem cells. While safety has been consistently demonstrated, particularly with autologous transplants, sustained curative benefit has not been consistently obtained. Allogenic transplants generally have major issues for continual immunosuppression to prevent rejection of grafted cells. In some cases, the benefit of cell therapy is through unidentified trophic effects of transient grafted cells. Nevertheless, progress for therapeutic benefit for patients is increasing and there is clear merit for using stem cells as delivery vehicles for correcting genetic mutations that cause severe disease phenotypes. Increasingly, new stem cell types are being explored and both neural and pluripotent stem cells (embryonic stem cells) are under study in early Phase I/II trials. It is too early to predict the outcome of these trials at present but early observations of patients indicate that they do appear to be safe. Recent studies using induced pluripotent stem cells (iPSCs) have shown sizeable genetic and epigenetic abnormalities in these cells and there is now a clear need to determine the biological significance of those changes before iPSCs are taken to clinical trials [41,42]. A strong indication of the confidence in the cell therapy field is the increasing participation of the large pharmaceutical companies in stem cell therapies [43]. Strong funding from organizations such as the Californian Institute for Regenerative Medicine and their collaborating partners worldwide is likely to rapidly expand new clinical trials in the next few years.

## List of abbreviations

ALS: amyotrophic lateral sclerosis; ASCs: adipose derived stem cells; EPCs: endothelial progenitor cells; G-CSF: granulocyte-colony stimulating factor; GVHD: graft versus host disease; hESCs: human embryonic stem cells; HSCs: hematopoietic stem cells; iPSCs: induced pluripotent stem cells; LDL: low-density lipoprotein; MSCs: mesenchymal stem cells; NSCs: neural stem cells; PMD: Pelizaeus-Merzbacher disease.

## Competing interests

CIRM's governing board recently voted to provide $25 million in loans to Geron to support the spinal cord injury trial noted in this review.

## Authors' contributions

AT was lead writer and editor with editorial assistance from DG and data gathering from GL and RGT. All authors read and approved the final manuscript.

## Pre-publication history

The pre-publication history for this paper can be accessed here:

http://www.biomedcentral.com/1741-7015/9/52/prepub
